# Preparation and electrochemical performances of carbon sphere@ZnO core-shell nanocomposites for supercapacitor applications

**DOI:** 10.1038/srep40167

**Published:** 2017-01-06

**Authors:** Xuechun Xiao, Bingqian Han, Gang Chen, Lihong Wang, Yude Wang

**Affiliations:** 1 Yunnan Province Key Lab of Micro-Nano Materials and Technology, Yunnan University, 650091 Kunming, People’s Republic of China; 2 School of Materials Science and Engineering, Yunnan University, 650091 Kunming, People’s Republic of China; 3 Department of Physics, Yunnan University, 650091 Kunming, People’s Republic of China

## Abstract

Carbon sphere (CS)@ZnO core-shell nanocomposites were successfully prepared through facile low-temperature water-bath method without annealing treatment. The morphology and the microstructure of samples were characterized by transition electron microscopy (TEM), X-ray diffraction (XRD) and X-ray photoelectron spectroscopy (XPS), respectively. ZnO nanoparticles with several nanometers in size decorated on the surface of the carbon sphere and formed a core-shell structure. Electrochemical performances of the CS@ZnO core-shell nanocomposites electrodes were investigated by cyclic voltammetry (CV) and galvanostatic charge/discharge (GDC). The CS@ZnO core-shell nanocomposite electrodes exhibit much larger specific capacitance and cycling stability is improved significantly compared with pure ZnO electrode. The CS@ZnO core-shell nanocomposite with mole ratio of 1:1 achieves a specific capacitance of 630 F g^−1^ at the current density of 2 A g^−1^. Present work might provide a new route for fabricating carbon sphere and transition metal oxides composite materials as electrodes for the application in supercapacitors.

Supercapacitors have attracted much attention for their high power density, long cycle life and green environmental protection^1^,^2^. Hence, they are widely utilized in consumer electronics, memory back-up systems, hybrid electric vehicles and industrial power management^3^,^4^. There are two widely accepted mechanisms currently for supercapacitors, i.e. electric double-layer capacitors (EDLCs) and pseudocapacitors. Charge could be stored by EDLCs electrostatically by using charge accumulation induced by electrostatic force on electrode materials’ surface. The EDLCs electrode materials are mainly carbon materials such as carbon sphere, activated carbon, carbon nanotubes (CNTs), reduced graphene and so on^5^,^6^. The pseudocapacitors, however, rely on the fast and reversible redox reaction between electrolyte ions and electroactive materials, which possess higher capacitance and superior energy density than EDLCs^7^. The pseudocapacitors electrode materials are primarily conducting polymers^8^,^9^ and transition metal oxides/hydroxides^10^,^11^,^12^. Considering their higher specific capacitance obtained from the Faradaic electrochemical reactions occurring on the surface of electrode materials, transition metal oxides have gained significant interest as active electrode materials for supercapacitor.

Among those transition-metal oxides having been studied comprehensively, zinc oxide (ZnO) is a suitable candidate for supercapacitor applications because of its good electrochemical activity, low cost as a raw material, and environmental friendliness^13^,^14^. Unfortunately, there is an obstacle of the zinc oxide’s applicability set by its low rate capability and poor repeatability during cycling in the process of developing ZnO as a promising supercapacitor electrode material^15^. However, these limitations may be overcome by using various composites consisting of carbon material and zinc oxide as electrode materials for electrochemical capacitor. Benefits of both the Faradaic capacitance of the metal oxide and the double layer capacitance of the carbon materials with large specific surface areas, which would in turn improve the capacitance and cycling stability^16^,^17^,^18^. A lot of researches focused on ZnO/carbon materials composite electrodes for supercapacitors. Zhang *et al*.^16^ synthesized ZnO/carbon nanotube composite, which achieved specific capacitances of 323.9 F g^–1^. D. Kalpana *et al*.^17^ fabricated ZnO/carbon aerogel composite electrodes with a very high specific capacitance of 500 F g^–1^ in 6 M KOH solution. Li *et al*.^18^ applied ZnO/activated carbon composite as the electrodes and obtained specific capacitances of 117.4 F g^–1^. Li *et al*.^14^ and Lu *et al*.^19^ prepared ZnO/graphene composite and acquired specific capacitances of 554.23 and 146 F g^–1^, respectively. However, to the best of our knowledge, no studies have been done using carbon sphere to produce the ZnO/carbon sphere composites as electrodes for supercapacitors. In addition, the core-shell structure could not only guarantee stable structure and favorable kinetics, but also provide high surface area and which ought to increase the amount of active sites on the surface of materials, which ensures the devices to possess satisfied electrochemical performance^20^. Hydrothermal method is one of the most important technologies in preparing CS@ZnO core-shell nanocomposites^21^. However, special apparatus (Teflon lined autoclave) and high reaction temperature (generally above 120 °C) and pressure energy-consuming procedures are needed. Thus it is of great importance to explore cheap and mild synthetic strategies to obtain CS@ZnO core-shell nanocomposites for supercapacitor electrode materials. To achieve this goal, in the present work we study the CS@ZnO core-shell nanocomposites prepared by a low-temperature and simple synthesis process.

Herein, we report a facile low-temperature water-bath method to fabricate CS@ZnO core-shell nanocomposites without annealing treatment, which possess stable core-shell structure. The reaction temperature required for the synthesis was only 60 °C and with other annealing treatment. The route is also expected to be capable to prepare a variety of CS@transition-metal oxides. The achieved CS@ZnO core-shell nanocomposites were used as electrode materials for supercapacitors. The electrochemical performances were studied in terms of specific capacitance and cycling stability in detail.

## Results and Discussion

**Figure 1:**
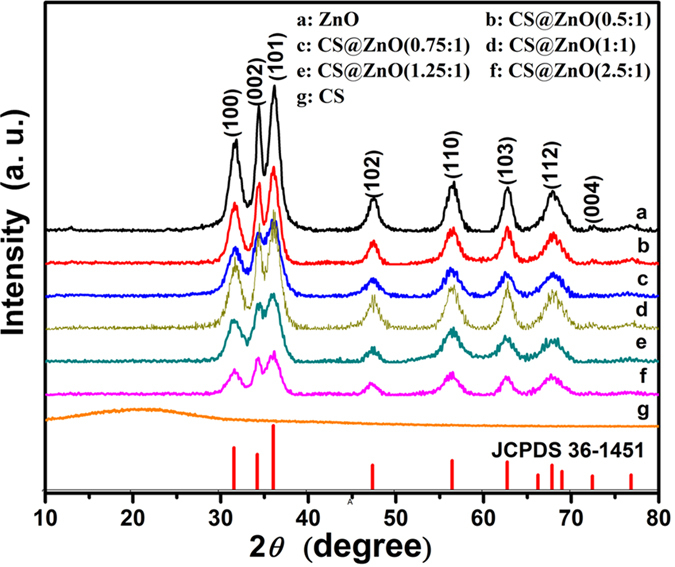
X-ray diffraction patterns of CS@ZnO core-shell nanocomposites with different molar ratio: (**a**) 0, (**b**) 0.5:1, (**c**) 0.75:1, (**d**) 1:1, (**e**) 1.25:1, (**f**) 2.5:1.

XRD patterns of the as-synthesized CS@ZnO core-shell samples with various mole ratios are shown in Fig. 1. It can be seen that the samples have the similar XRD patterns, which can be well-indexed to wurtzite structure of ZnO (hexagonal space group: P6_3_mc, lattice constants a = b = 3.25 Å, c = 5.207 Å, JCPDS No. 36–1451). There is no diffraction peak originating from the carbon species in the XRD spectrum although with the carbon composite ratio increasing, probably due to the composite carbon sphere is amorphous phase and its diffraction peak is broad peak (curve g in Fig. 1). Because of the intensity of the broad peak is weak and disappear after the carbon sphere composited with ZnO, and the diffraction peaks of the nanocomposite mainly manifest the characteristic diffraction peaks of ZnO. The characteristics of the XRD pattern demonstrate that carbon sphere does not change the crystal structure of ZnO. The broadening of the peaks means a small crystallite size. The crystallite size (*D*) of the as-synthesized samples were estimated from the (110) plane diffraction peak (2*θ* = 56.6°) by using the Scherrer equation: *D* = 0.9*λ*/(*β*cos *θ*), where *λ* is the wavelength of X-ray (1.54056 Å), *θ* is the Bragg’s diffraction angle, and *β* is the true half-peak width of the X-ray diffraction lines. The estimated crystallite sizes are about 7 nm.

**Figure 2:**
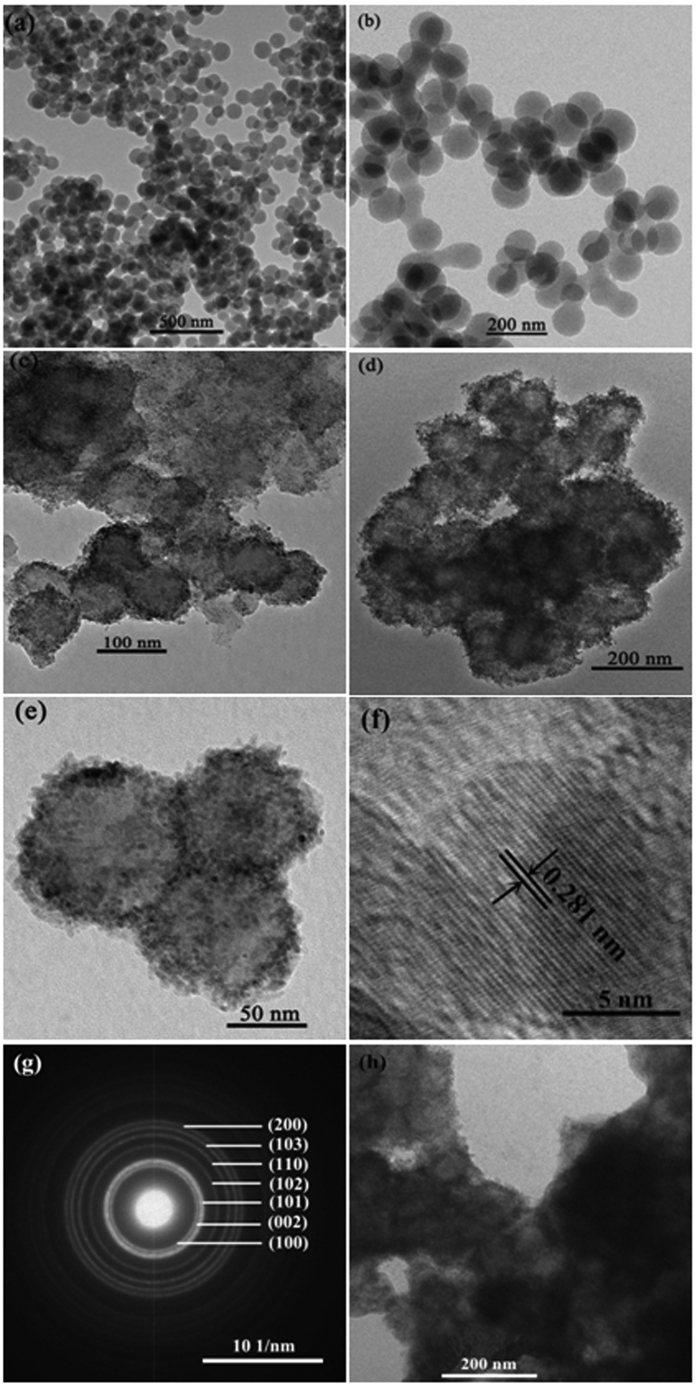
The TEM images of carbon spheres and CS@ZnO core-shell nanocomposites with different ratio: (**a**,**b**) are the whole and partial images of carbon spheres, (**c**) CS@ZnO core-shell nanocomposites (0.75:1), (**d**,**e**) are the whole and partial images of CS@ZnO core-shell nanocomposites (1:1), (**f**,**g**) are the HRTEM and SAED images of ZnO in the CS@ZnO core-shell nanocomposites (1:1),(**h**) CS@ZnO core-shell nanocomposites (2.5:1), respectively.

The morphologies of the carbon sphere and the CS@ZnO core-shell composites were investigated using TEM. Figure 2(a,b) show the morphology and microstructure of carbon sphere sample. As observed in Fig. 2(a), the perfect carbon sphere can be uniformly prepared by hydrothermal method. In the magnified TEM image as shown in Fig. 2(b), a majority of carbon sphere with homogeneous size (about 106.7 nm) and smooth surface can be clearly demonstrated. The carbon spheres were used as templates and coated with ZnO nanoparticles layer. Figure 2(c) show typical TEM image of sample with 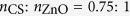, which indicates that ZnO nanoparticles decorated on the surface of the carbon sphere and the CS@ZnO core-shell nanocomposite particle exhibit integrally sphere shape. In addition, it can be seen from the Fig. 2(c) that a part of ZnO nanoparticles scattered from the carbon sphere and piled up together, probably due to the low content of carbon sphere in the ZnO sample. With the content of carbon sphere increasing to 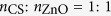, there are no ZnO nanoparticles scattering from the carbon spheres (Fig. 2(d)). The interface between ZnO shell and carbon core can be seen clearly as shown in Fig. 2(e). All ZnO nanoparticles uniformly grow on the surface of the carbon spheres and the composite nanoparticles form stable core-shell structure. From the HRTEM images (Fig. 2(f)) of the CS@ZnO core-shell nanocomposites (1:1), it can be clearly seen that the diameter of ZnO nanoparticles is estimated 5–10 nm and their crystalline structures can be observed clearly from the lattice fringes of the ZnO (100) plane with an interplanar distance of about 0.281 nm. The ZnO nanoparticles were crystalline, further confirmed by the selected-area electron diffraction(SEAD) analysis (Fig. 2(g)).The Fig. 2(h) shows typical TEM image of sample in 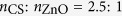, which indicates that the agglomeration becomes very serious with the increase of carbon spheres. The TEM analysis indicates that the sample in 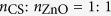 possessed uniform spherical structure, which enable effective electrolyte access into the electrode and is favorable for better supercapacitor performance.

**Figure 3:**
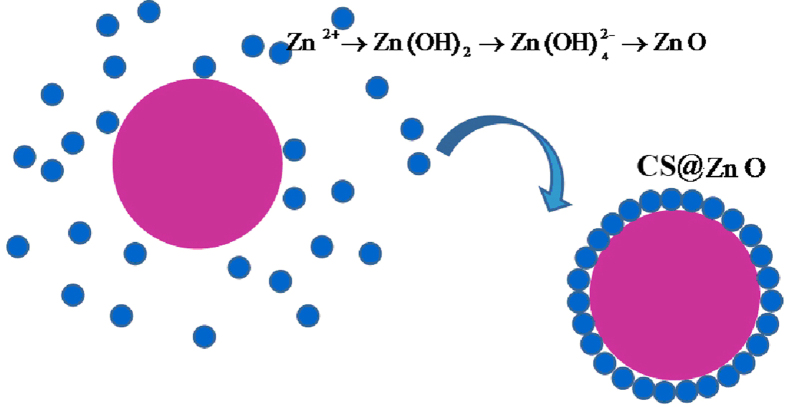
A schematic illustration of the formation process of CS@ZnO nanocomposites.

A possible forming mechanism could be illustrated on the basis of the series of experimental data. The schematic diagram of the forming mechanism of CS@ZnO core-shell nanocomposites is presented in Fig. 3. Carbon spheres were obtained by the carbonization of glucose monomers, which was attributed to the cross-linking induced by intermolecular dehydration of macromolecules. The surface of as-prepared carbon spheres has s distribution of residual hydroxyl and carbonyl groups^22^. The morphology evolution process can be explained based on Ostwald ripening^23^. First, numerous ZnO generate small crystallites nucleate under the water bath condition. Then, the small crystallites were absorbed on the surface of carbon spheres without any modifications to minimize their surface energies by an oriented attachment. Finally, CS@ZnO core-shell nanocomposites which aggregated ZnO nanoparticles formed the extrinsic layer of carbon spheres were obtained. Impedance match with free space was achieved by the micro-bilayer of the obtained composites^21^.

**Figure 4:**
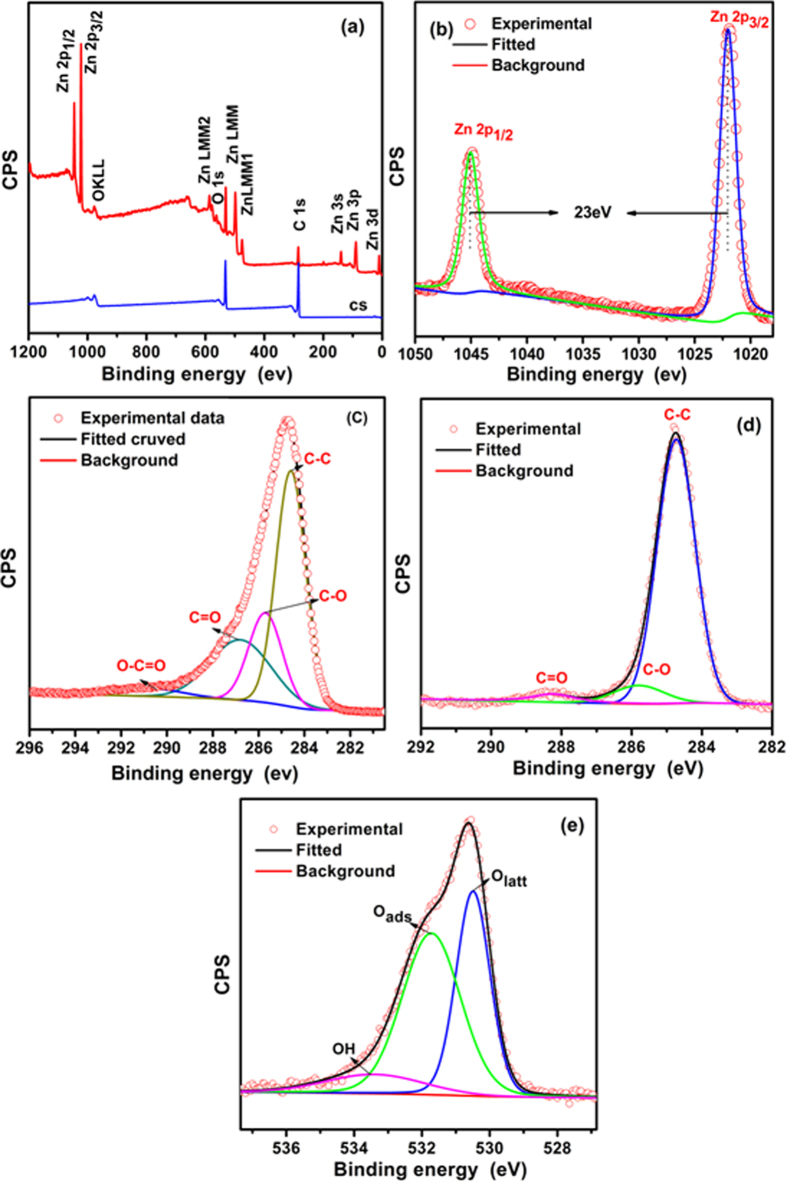
XPS spectra of CS and CS@ZnO core-shell nanocomposites (1:1): (**a**) survey spectrum, (**b**) Zn 2p, (**c**) C 1 s of CS, (**d**) C 1 s of CS@ZnO core-shell nanocomposites (1:1), (**e**) O 1 s of CS@ZnO core-shell nanocomposites (1:1).

The XPS measurements were carried out to obtain more detailed information about elemental compositions and chemical states of pure CS and CS@ZnO core-shell nanocomposites 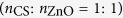, and the results are presented in Fig. 4. Compared to that of CS, the CS@ZnO core-shell nanocomposites not only exhibits O 1 s and C 1 s peaks, but also exhibits the Zn 2p_1/2_ and Zn 2p_3/2_ peaks, which confirm the presence of Zn species in the composite (Fig. 4(a)). The strong resolution Zn 2p spectrum is presented in (Fig. 4(b)), of which two strong peaks at 1022.02 and 1045.05 eV can be clearly seen, corresponding to the binding energy of Zn 2p_3/2_ and Zn 2p_1/2,_ respectively, indicating the presence of Zn^2+^ in the ZnO wurtzite structure^24^. It is observed that there is an energy separation of 23 eV between the Zn 2p_3/2_ and Zn 2p_1/2_ peaks, which is in agreement with an earlier report on ZnO^25^. The high resolution C1s spectrum of pure CS (Fig. 4(c)) reveals that there are four main components arising from C-C, C-O, C=O and O-C=O groups. The strong peak at 284.62 eV can be assigned to the C-C bond of carbon sphere, and the broad peaks at 285.73, 286.79 and 290.04 eV to C-O, C=O and O-C=O bonds, which indicates the substantial introduction of oxygen-containing functional groups on the carbon sphere, which is ascribed to the carbonization of glucose monomers. Compared with the CS, the high resolution C1s spectrum of CS@ZnO (Fig. 4(d)) reveals that there are three main components arising from C-C, C-O and C=O groups, at 284.76 eV, 285.79 and 288.25 eV, respectively. In the spectrum recorded after water-bath reduction of the composite, the intensities of all of the C1 s peaks of the carbon atoms bound to oxygen, especially the C-O (epoxy and alkoxy) peak, decreased dramatically, revealing that most of the epoxide and hydroxyl functional groups were successfully removed, which makes the CS a good channel for electronic conductivity. The functional groups on the carbon sphere might further improve its electrochemical capacitance^25^. From the O1s spectrum (Fig. 4(f)), it can be seen the spectrum can be fitted to two gauss peaks which one is O_latt_ (530.51 eV) and the other one is O_ads_ (531.77 eV). O_latt_ is defined as oxygen ions in the crystal lattice, while O_ads_ is the absorbed oxygen ions in the oxygen deficient regions. This phenomenon indicates that there is not only lattice oxygen but also adsorption oxygen in the sample^26^. These functional groups are beneficial to capacitive performance because they can contribute to additional pseudocapacitance and improve the wettability between electrode and electrolyte^27^.

**Figure 5:**
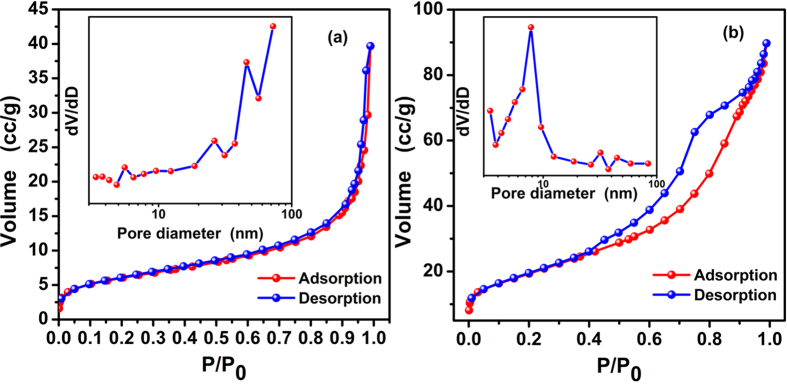
Nitrogen adsorption-desorption isotherm of (**a**) pure ZnO and (**b**) CS@ZnO core-shell nanocomposites (1:1). Insert is the pore-size distribution calculated by the BJH method from the desorption branch of pure ZnO and CS@ZnO core-shell nanocomposites (1:1), respectively.

The surface area and pore size distribution of pure ZnO and the CS@ZnO core-shell nanocomposites (1:1) are measured by nitrogen adsorption/desorption method at 77 K. Figure 5(a,b) are N_2_ adsorption-desorption isotherms and corresponding pore size distribution plots (using the BJH calculation model) of pure ZnO and the CS@ZnO core-shell nanocomposites (1:1). As can be seen in Fig. 5, the ZnO and the CS@ZnO nanocomposites exhibit a type IV hysteresis loop and H3 hysteresis loop according to the IUPAC classification. From the pore size distribution curve (embedding figure in Fig. 5), it can be seen the size of the pores exhibits a strong peak at about 45.9 nm for the ZnO, while at about 7.96 for the CS@ZnO nanocomposites. It suggests that the CS@ZnO nanocomposites possess a number of mesoporous. The BET surface areas of pure ZnO and CS@ZnO core-shell nanocomposites (1:1) are examined to be 25.35 and 115.70 m^2^·g^−1^, respectively. These BET analysis indicated that CS@ZnO core-shell nanocomposites (1:1) possessed higher surface area, better mesoporous structure, which is suitable for better supercapacitor performance.

**Figure 6:**
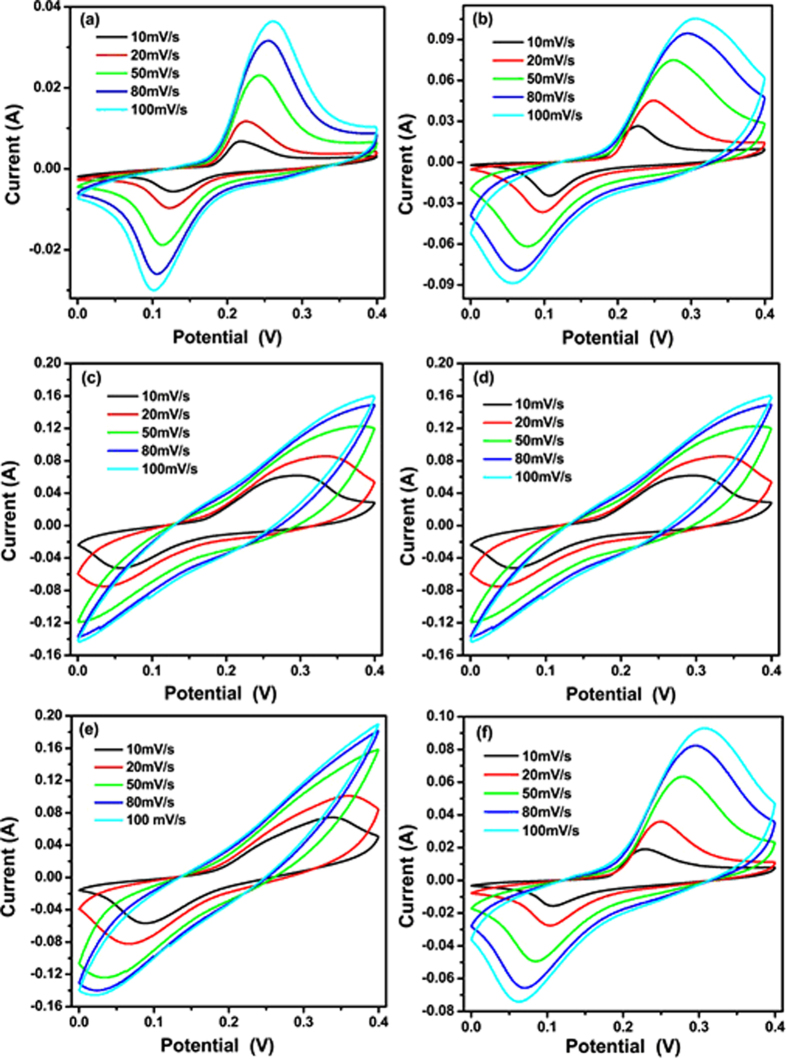
CV curves of CS@ZnO core-shell nanocomposites with different molar ratio: (**a**) ZnO nanoparticles, (**b**) CS@ZnO core-shell nanocomposites ratio of 0.5:1, (**c**) 0.75:1, (**d**) 1:1, (**e**) 1.25:1 and (**f**) 2.5:1, respectively.

The electrochemical properties of CS@ZnO core-shell nanocomposites electrodes were manufactured and studied in 6 M KOH solution by cyclic voltammetry (CV) and charge-discharge measurements. Figure 6 shows the CV curves of different proportion CS@ZnO core-shell nanocomposites electrodes with the potential window of 0–0.4 V at a scan rate of 10–100 mV s^–1^. The CV shapes clearly reveal that the capacitance characteristic of CS@ZnO core-shell nanocomposites is a typical pseudocapacitance. One pair of redox peaks are observed in the CV curve, caused by redox reactions of ZnO. This redox process is mainly governed by the intercalation and deintercalation of K^+^ from electrolyte into ZnO^28^,^29^:


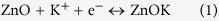


**Figure 7:**
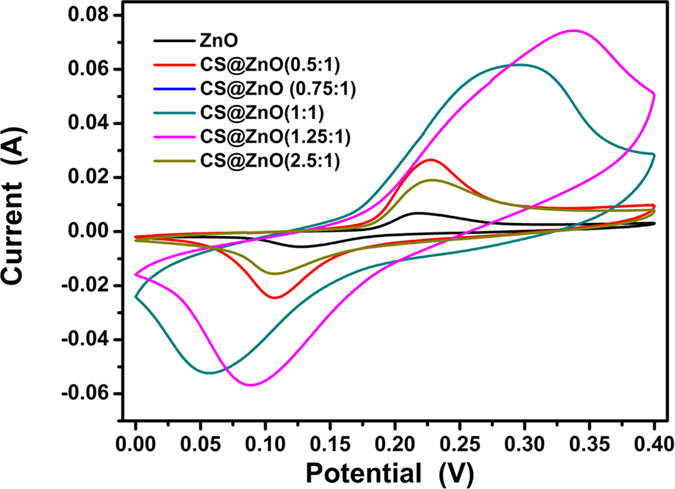
Comparison of CV curves of CS@ZnO core-shell nanocomposites with different molar ratio at a scan rate of 10 mV·s^–1^.

Figure 7 shows the comparison of the CV curves of CS@ZnO core-shell nanocomposites with different molar ratio at a scan rate of 10 mV s^–1^, from which we can learn that the area under the CV curve is increased firstly and then decreased with increasing gradually the carbon content, the enclosed area reaches maximum at the 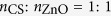. It is well-known that the specific capacitance is proportional to the area of the CV curve^30^. Therefore, the order of the specific capacitance of the CS@ZnO core-shell nanocomposites electrodes prepared by different molar ratio, which can be further verified by the following charge-discharge measurements.

**Figure 8:**
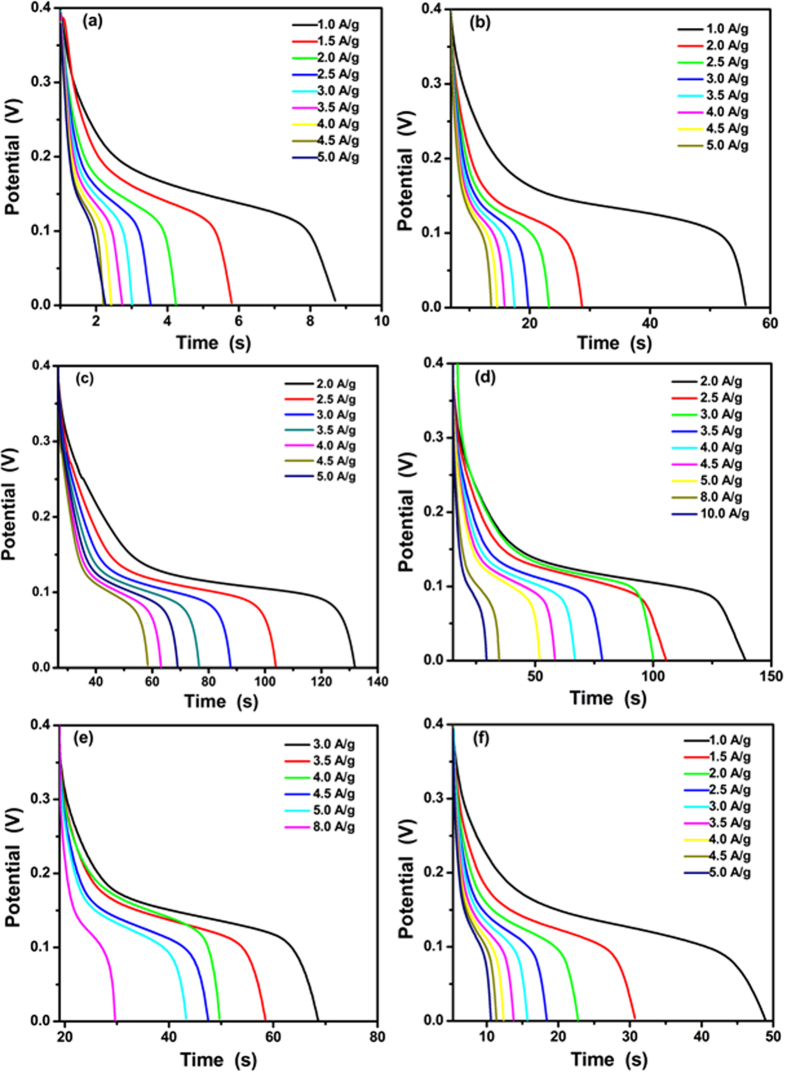
Galvanostatic curves of CS@ZnO core-shell nanocomposites with different molar ratio: (**a**) ZnO nanoparticles, (**b**) CS@ZnO core-shell nanocomposites ratio of 0.5:1, (**c**) 0.75:1, (**d**) 1:1, (**e**) 1.25:1 and (**f**) 2.5:1, respectively.

**Figure 9:**
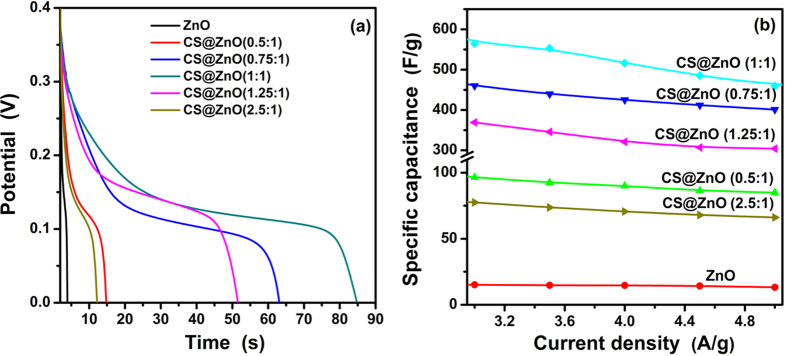
(**a**) comparison of galvanostatic charge and discharge curves of CS@ZnO core-shell nanocomposites with different molar ratio at a current density of 3 A·g^–1^, (**b**) the specific capacitance for CS@ZnO core-shell nanocomposites with different molar ratio versus current density.

Figure 8 manifest the galvanostatic discharge curves of the CS@ZnO core-shell nanocomposites electrodes prepared by different molar ratio at various current densities with a potential range of 0–0.4 V. The nonlinear discharge curves and the voltage plateaus match well the peaks observed from the CV curves, which further verifies the pseudocapacitance behavior of the CS@ZnO core-shell nanocomposites electrodes. The specific capacitances (*C*, (F g^−1^)) were calculated according to the following equations:


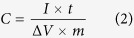


where *I* (A) is the constant discharge current, *t* (s) is the discharging time, *m* (g) is the total mass of active materials in both electrodes, ∆*V* (V) is the operating voltage window obtained from the discharge curve excluding the voltage drop. The calculated specific capacitance values for the pure ZnO, CS@ZnO (0.5:1), CS@ZnO (0.75:1), CS@ZnO (1:1), CS@ZnO (1.25:1) and CS@ZnO (2.5:1) at a current density of 3 A g^–1^ are 15.14, 96.49, 459.9, 564.41, 369.1 and 77.52 F g^–1^, respectively. The CS@ZnO (1:1) electrode achieves the highest specific capacitance among all shown in (Fig. 9(a)), and with the 37-fold increase of the pure ZnO. This is due to the higher BET specific surface areas of CS@ZnO (1:1) calculated to be 115.70 m^2^ g^–1^ (pure ZnO, 25.35 m^2^ g^–1^), which increases electrolyte/electrode contact areas and hence provides more active sites for fast faradaic redox reactions. From Fig. 9(b), the specific capacitances of the CS@ZnO (1:1) electrodes are calculated to be 630, 564.41, 553, 516, 485 and 459 F g^–1^ at current densities of 2.0, 3.0, 3.5, 4.0, 4.5 and 5 A g^–1^, respectively. On the other hand, as listed in Table 1, the specific capacitance value of the CS@ZnO core-shell nanocomposites electrodes are the highest than those reported in literatures^14^,^16^,^17^,^18^,^19^,^25^,^28^,^29^,^31^,^32^. The capacity retention rate (compare to 3 A g^–1^) is 81.04% at the current density of 5 A g^–1^. The CS@ZnO (1:1) electrode manifests higher capacitance and better rate capability, which is in agreement with the result of the CV curves.

**Figure 10:**
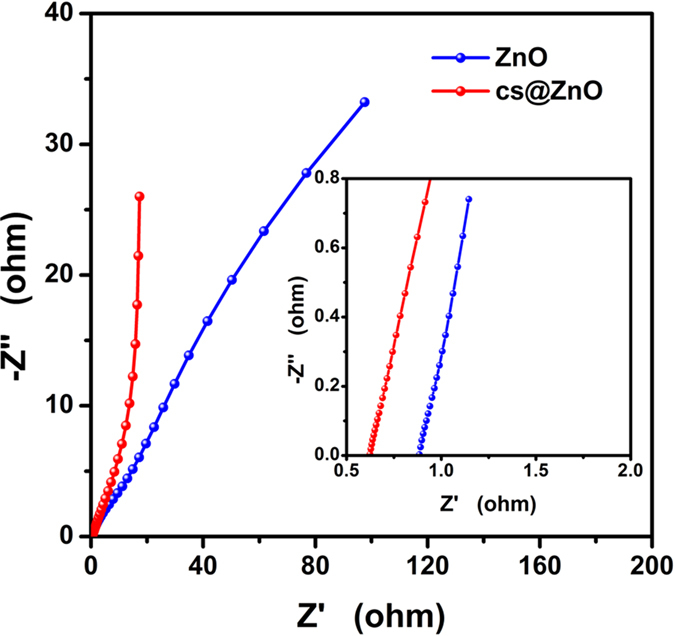
Nyquist plot of the pure ZnO and CS@ZnO core-shell nanocomposites (1:1).

In order to discuss the transport characteristics of the charge carries in sample of CS@ZnO(1:1) and bare ZnO electrodes, the electrochemical impendence spectroscopy (EIS) was also complemented at open circuit potential over the frequency range 0.01to 100,000 Hz. With the EIS spectra in Fig. 10, both Nyquist plots shape of a semicircle at high frequency regions and an inclined line in low frequency regions. At high frequencies, the point intersecting with the real axis exhibits the internal resistance (*R*_s_), including the intrinsic resistance of the electrode active material, bulk resistance of electrolyte, and contact resistance at the interface between active material and current collector interface. Besides, the diameter of the semicircle corresponds to the interfacial charge transfer resistance (*R*_ct_). At the lower frequency, the linear line is related to the Warburg impedance or diffusion resistance, and the unit of W is S sec^1/2^/cm^2^, so higher value indicating lower impedance^33^. From the Fig. 10, it can be seen that the slope of the straight line for the CS@ZnO(1:1) nanocomposite electrode is larger than that of the ZnO electrode, That is to say the CS@ZnO(1:1) nanocomposites have a better ions diffusion in the electrode. At high frequencies, the point intersecting with the real axis of the CS@ZnO(1:1) nanocomposite electrode is smaller than of the ZnO electrode. which means that the conduction of the CS@ZnO nanocomposites was improved by compositing the carbon sphere with good electrical conductivity. The better ion diffusion can be ascribed to the morphology of the CS@ZnO nanocomposites possessed core-shell structure as well as larger specific surface area, which are beneficial to the ion diffusion, and the specific capacitance was improved.

**Figure 11:**
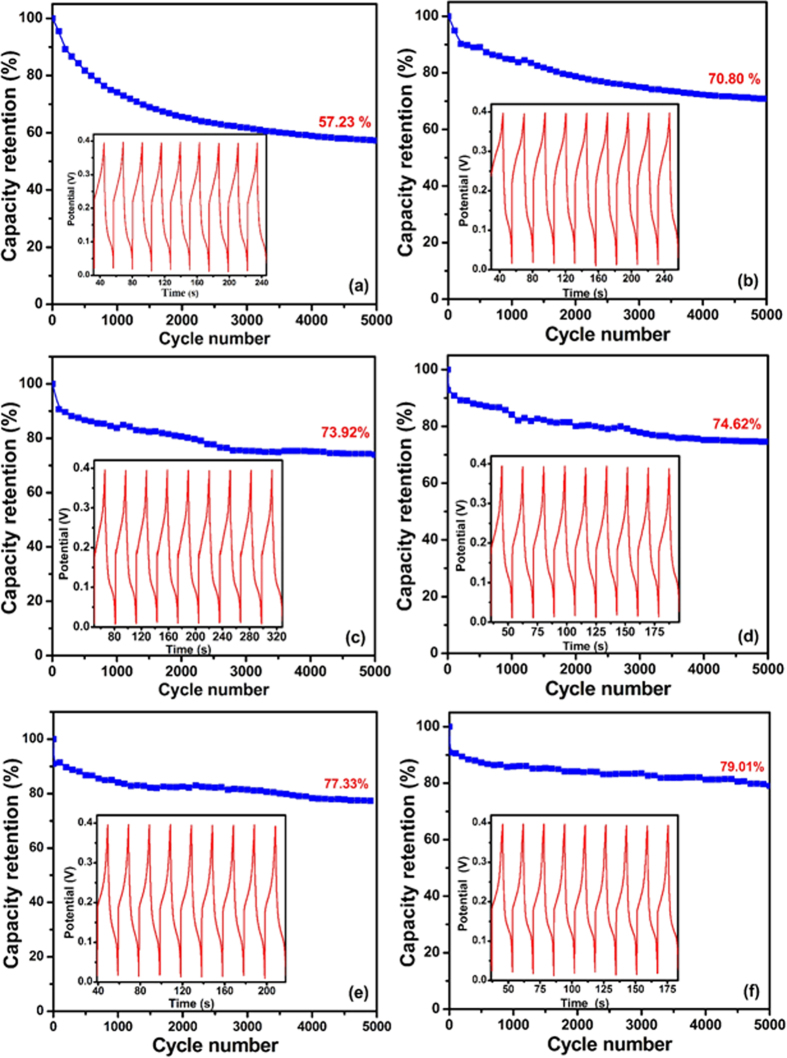
The specific capacitance for CS@ZnO core-shell nanocomposites with different molar ratio versus cycle number: (**a**) ZnO nanoparticles, (**b**) CS@ZnO core-shell nanocomposites ratio of 0.5:1, (**c**) 0.75:1, (**d**) 1:1, (**e**) 1.25:1 and (**f**) 2.5:1, respectively.

For the supercapacitor electrode, the cycle stability is an important evaluation parameter. The cycling performance of the CS@ZnO core-shell nanocomposites electrodes with different molar ratio were further conducted in the potential range of 0–0.4 V in 6 M KOH solution for 5000 cycles. As shown in Fig. 11, the capacitance of all the CS@ZnO core-shell nanocomposites electrode exhibit lowest degradation during the test, which imply that they have excellent long-term recycling capability. The capacitance of the electrodes retained over 70% of the initial capacitance after 5000 cycles. In contrast, the pure ZnO electrode exhibits the lowest capacity retention and the capacitance declined to 57.23% of the initial capacitance after 5000 cycles. It is speculated that the excellent stability might be mainly due to the existence of stable carbon sphere with high chemical and thermal stability acting as temple and channel of the charge transferring. Meanwhile the core-shell structure of CS@ZnO nanocomposites is very stable, and the cycle stability of the electrode active material is gradually enhanced with the increase of carbon sphere ratio.

The above results of CS@ZnO core-shell nanocomposites exhibit excellent electrochemical performance because of the positive synergy of the faradic capacitance of the zinc oxide and the double layer capacitance of the carbon sphere. Firstly, the conductivity of the CS@ZnO composites was enhanced by the addition of carbon sphere with high conductivity, which is very important to the rate capability and power density under the high current density. Secondly, the CS@ZnO (1:1) core-shell nanocomposites have a relatively larger accessible surface area of 115.69 m^2^ g^–1^ than the pure ZnO with the surface area of 25.35 m^2^ g^–1^. Through combination with carbon sphere with large specific surface area, which can increase the effective contact area between the electrolyte and electrode materials, and improve the transmission of electronic and the diffusion of ion, thus increase the specific capacitance. In the end, the specific core-shell structure of the CS@ZnO nanocomposite provide more channels for ion transport, which can improve the electrochemical performance. Form the TEM morphology analysis, all ZnO nanoparticles uniformly grow on the surface of the carbon sphere and the composite nanoparticles form stable core-shell structure only the mole ratio of sample in 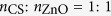. Hence, it can be explained that the CS@ZnO (1:1) electrodes manifests higher capacitance and better rate capability.

## Conclusions

In summary, the CS@ZnO core-shell nanocomposites at different molar ratios were successfully synthesized through facile low-temperature water-bath method without annealing treatment. The characteristics of the XRD patterns demonstrate that carbon sphere does not change the crystal structure of ZnO. ZnO nanoparticles decorated on the surface of the carbon sphere and the CS@ZnO core-shell nanocomposites exhibit integrally sphere shape. The BET indicated that the CS@ZnO core-shell nanocomposites have the higher specific surface area than ZnO nanoparticles. The samples were used as electroactive materials for supercapacitor. The results indicate that the CS@ZnO core-shell nanocomposites can improve electrochemical properties, including the specific capacitance and the cycle stability. In detail, the CS@ZnO core-shell nanocomposite (at the mole ratio of 1:1) electrode exhibits the maximum specific capacitance as high as 630 F g^−1^ at the current density of 2 A g^−1^, which is 32-fold higher than that of the pure ZnO electrode prepared through the same method. Furthermore, it significantly improves cycling stability compared to the pure ZnO electrode, and the specific capacitance retention of 70.80% after 5000 cycles. The properties of CS@ZnO core-shell nanocomposites exhibit a lot of development space in supercapacitor applications.

## Methods

All the reagents used in the experiments were purchased from commercial sources of analytical grade and used without further purification.

### Preparation of carbon spheres

Carbon spheres were prepared by a facile hydrothermal method. In a typical procedure, a certain quality of glucose powder was dissolved into distilled water with stirring to get 0.5 M solution. The solution was transferred to a Teflon-lined. Subsequently, the Teflon bottle with this mixture was held in a stainless steel vessel autoclave. Hydrothermal reaction was conducted at 180 °C for 10 h in an oven. The autoclave was cooled down to room temperature. The resulting products were centrifuged and thoroughly washed with distilled deionized water and ethanol three times, respectively. Finally, brown powders were obtained after being dried at 60 °C overnight.

### Preparation of CS@ZnO core-shell nanocomposites

CS@ZnO core-shell nanocomposites were prepared by a simple low temperature water-bath method. In a typical synthesis of precursors, 0.395 g Zn(CH_3_CO_2_)_2_·2H_2_O were dissolved into 50 mL absolute ethyl alcohol with stirring until a homogenous solution (A) was obtained. The as-prepared brown carbon sphere (CS) with a certain mole ratio 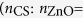 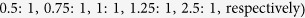 was dispersed into 30 mL absolute ethyl alcohol with violently stirring. The dispersed solution (B) was dripped into the previous homogenous solution (A) to obtain the mixture solution (C) with maintaining stirring under water-bath conditions at a temperature of 60 °C for 1 h. 0.6 g NaOH were dissolved into 50 mL absolute ethyl alcohol with stirring until a homogenous solution (D) was obtained. Next, the NaOH solution (D) was added dropwise into the got solution (C) with maintaining stirring under water-bath conditions at a temperature of 60 °C for 2 h. The resulting products were centrifuged, and the as-prepared CS@ZnO core-shell nanocomposites were thoroughly washed with distilled deionized water and ethanol three times, respectively, and then dried at 60 °C overnight.

### Characterization of CS@ZnO core-shell nanocomposites

The crystal orientation was investigated by using a Rigaku D/max-3B diffractometer with Cu Kα radiation (*λ* = 1.54056 Å). The data were collected between scattering angles (2*θ*) from 10° to 90° in step of 0.02°. TEM images of the microstructure of core-shell nanocomposites were also carried out by (JEOL JEM-2100) at an acceleration voltage of 200 kV. The samples for TEM were prepared by dispersing the final powders in ethanol; this dispersing was then dropped on carbon-copper grids. XPS was carried out at room temperature in a PHI 5500 spectrometer with polychromatic Al/Mg-Kα X-ray source. During XPS analysis, Al K*α* X-ray beam was adopted as the excitation source and power was set to 250 W. Vacuum pressure of the instrument chamber was 1 × 10^−7^ Pa as read on the panel. Measured spectra were decomposed into Gaussian components by a least-square fitting method. Bonding energy was calibrated with reference to C1s peak (285.0 eV). The BET surface areas were measured by nitrogen adsorption/desorption using a NOVA2200e gas sorption analyzer (Quantachrome Corp.).

### Electrode preparation and electrochemical characterization

To investigate the electrochemical properties, CS@ZnO core-shell nanocomposites electrodes were constructed by mixing the active materials, acetylene black and Poly(vinyldiFluoride) (PVDF, in N-methyl-pyrrolidone solvent with solid content of 10 g L^−1^), in the weight ratio 85:10:5. The mixture was prepared as homogeneous slurry in a small amount of ethanol solution and pressed onto the nickel foam (which was first pretreated successively with diluted hydrochloric acid and absolute ethanol to ensure a clean surface). The pasted nickel electrodes were dried at 120 °C for 24 h, and followed by pressing under a pressure of 8 MPa. The area of working electrode is about 1 cm^2^ and the weight of the active material in electrode is about ~5 mg cm^−2^. All electrochemical measurements were performed using CHI 600E electrochemical workstation (ChenHua Instruments, Shanghai) in a three-electrode mode, including a platinum foil as counter electrode and a standard calomel electrode as reference electrode. The experiments were operated at room temperature in a 6 M KOH aqueous electrolyte. The electrochemical properties of the products were investigated with CV and GDC tests.

## Additional Information

**How to cite this article:** Xiao, X. *et al*. Preparation and electrochemical performances of carbon sphere@ZnO core-shell nanocomposites for supercapacitor applications. *Sci. Rep.* **7**, 40167; doi: 10.1038/srep40167 (2017).

**Publisher's note:** Springer Nature remains neutral with regard to jurisdictional claims in published maps and institutional affiliations.

## Figures and Tables

**Table 1:** Comparisons of the specific capacitance with the various carbon materials/ZnO composites.

Materials	Test system	Specific capacitance	Scan rate or current density	Electrolyte	Ref.
Graphene-ZnO nanocomposite	three-electrode mode	146 F g^–1^	2 mV s^–1^	1 M KCl	19
Activated carbon/ZnO composites	two-electrode mode	117.4 F g^–1^	0.5 A g^–1^	6 M KOH	18
ZnO/carbon aerogel composites	three-electrode mode	500 F g^–1^	100 mA cm^–1^	6 M KOH	17
Graphene-ZnO composite film	three-electrode mode	11.3 F g^–1^	100 mV s^–1^	1 M KCl	28
Carbon nanotube-ZnO nanocomposite	two-electrode mode	323.9 F g^–1^	50 mV s^–1^	1 M KCl	16
3D ZnO/rGO/ZnO sandwich-structured	three-electrode mode	275 F g^–1^	5 mV s^–1^	1 M Na_2_SO_4_	31
ZnO/reduced graphene oxide (RGO) nanocomposite films	three-electrode mode	95 F g^–1^	10 mV s^–^1	1 M Na_2_SO_4_	29
3D graphene ZnO nanorods composite networks	three-electrode mode	554.23 F g^–1^	5 mV s^–1^	1 M KOH	14
Carbon nanotube (CNT) and zinc oxide composite	three-electrode mode	126.3 F g^–1^	No giving	Gel polymer	32
Zinc oxide/activated carbon nanofiber composites	three-electrode mode	178.2 F g^–1^	No giving	6 M KOH	25
CS@ZnO core-shell nanocomposites	three-electrode mode	630 F g^–1^	2 A g^–1^	6 M KOH	**This work**
